# Proximal tubular epithelial insulin receptor mediates high-fat diet–induced kidney injury

**DOI:** 10.1172/jci.insight.143619

**Published:** 2021-02-08

**Authors:** Hak Joo Lee, Meenalakshmi M. Mariappan, Luke Norton, Terry Bakewell, Denis Feliers, Sae Byeol Oh, Andrew Donati, Cherubina S. Rubannelsonkumar, Manjeri A. Venkatachalam, Stephen E. Harris, Isabelle Rubera, Michel Tauc, Goutam Ghosh Choudhury, C. Ronald Kahn, Kumar Sharma, Ralph A. DeFronzo, Balakuntalam S. Kasinath

**Affiliations:** 1 Center for Renal Medicine, Division of Nephrology,; 2 Division of Diabetes, Department of Medicine,; 3 Department of Pathology, and; 4 Department of Periodontics, University of Texas Health, San Antonio, Texas, USA.; 5 Universite Cote d’Azur, CNRS – UMR-7370, Laboratoire de Physiomédecine Moléculaire, Nice, France.; 6 VA Research and; 7 Geriatric Research, Education and Clinical Center, South Texas Veterans Health Care System, San Antonio, Texas, USA.; 8 Joslin Diabetes Center and Harvard Medical School, Boston, Massachusetts, USA.

**Keywords:** Nephrology, Insulin signaling, Obesity

## Abstract

The role of insulin receptor (IR) activated by hyperinsulinemia in obesity-induced kidney injury is not well understood. We hypothesized that activation of kidney proximal tubule epithelial IR contributes to obesity-induced kidney injury. We administered normal-fat diet (NFD) or high-fat diet (HFD) to control and kidney proximal tubule IR–knockout (KPTIRKO) mice for 4 months. Renal cortical IR expression was decreased by 60% in male and female KPTIRKO mice. Baseline serum glucose, serum creatinine, and the ratio of urinary albumin to creatinine (ACR) were similar in KPTIRKO mice compared to those of controls. On HFD, weight gain and increase in serum cholesterol were similar in control and KPTIRKO mice; blood glucose did not change. HFD increased the following parameters in the male control mice: renal cortical contents of phosphorylated IR and Akt, matrix proteins, urinary ACR, urinary kidney injury molecule-1–to-creatinine ratio, and systolic blood pressure. Renal cortical generation of hydrogen sulfide was reduced in HFD-fed male control mice. All of these parameters were ameliorated in male KPTIRKO mice. Interestingly, female mice were resistant to HFD-induced kidney injury in both genotypes. We conclude that HFD-induced kidney injury requires renal proximal tubule IR activation in male mice.

## Graphical abstract

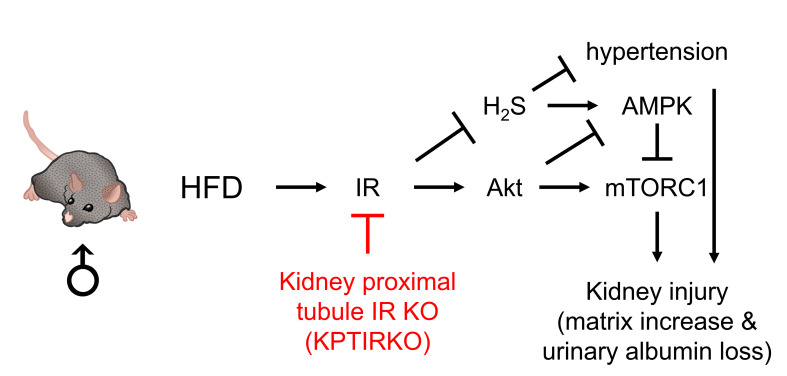

## Introduction

Insulin-resistant states, such as obesity, diabetes, and hypertension, are common causes of chronic kidney disease (CKD, refs. 1–3). Obesity predisposes to type 2 diabetes. Data from the CDC indicate that the prevalence of obesity in the US adult population is 39.8%, affecting nearly 93 million people, whereas it is 18.5% in youth (4). If the same trend continues, by 2030 nearly half of all adults will be obese (5).

Obesity causes kidney injury, leading to albuminuria, glomerular enlargement, focal and segmental glomerulosclerosis, and interstitial fibrosis (6). Compared with healthy adults, subjects with obesity carry a 2.8-fold and 6.3-fold higher risk for CKD and proteinuria, respectively, after adjusting for creatinine, uric acid, hypertension, HDL-cholesterol, and hyperglycemia (7). Studies on mechanisms through which obesity affects the kidney have assigned a role for dyslipidemia, adiponectin, angiotensin II, oxidative stress, hyperfiltration, immune activation, and lipotoxicity (8–14). In high-fat diet–induced (HFD-induced) obesity inhibition of AMP-activated protein kinase (AMPK) contributes to dyslipidemia, activation of Na-K-2Cl cotransporter, and hypertension (9, 15, 16). However, the critical events proximal to these pathways are not known. For instance, whether hyperinsulinemia, a key feature in obesity, plays a pathogenic role has not been directly examined.

Our suspicion that hyperinsulinemia could be involved in renal injury arose when we observed that renal cortical laminin accumulation occurred by nontranscriptional mechanisms in *db/db* mice with hyperinsulinemia, obesity, and type 2 diabetes (17). These nontranscriptional mechanisms could involve stimulation of mRNA translation of ambient laminin transcripts by increased levels of insulin, a powerful inducer of that ribosomal process. First, we established that insulin receptor (IR) was activated in the renal cortex of *db/db* mice (18). Second, studies in proximal tubular epithelial cells in vitro indicated that insulin rapidly drove laminin mRNA translation similar to high glucose (19–21). Finally, institution of a combined high-glucose and high-insulin clamp for 7 hours in normal rats reproduced mRNA translation pathways leading to laminin accumulation (22). This study left unanswered the question of the role of hyperinsulinemia independent of hyperglycemia in kidney injury. Thus, we employed the HFD model in this study, with hyperinsulinemia as the variable in the absence of hyperglycemia.

The goal of the present study was to test the hypothesis that renal proximal tubule epithelial IR activation contributes to obesity-induced kidney injury. We used kidney proximal tubule IR–knockout (KPTIRKO) mice with targeted deletion of the IR in the proximal tubule epithelial cell. Our findings reveal that activation of proximal tubular IR is required for obesity-induced kidney injury in mice.

## Results

### Characterization of KPTIRKO mice.

KPTIRKO mouse progeny grew normally. At 3 months of age, body weight and kidney weight of KPTIRKO mice were similar to those of the control mice (Supplemental Figure 1; supplemental material available online with this article; https://doi.org/10.1172/jci.insight.143619DS1).

**Figure 1:**
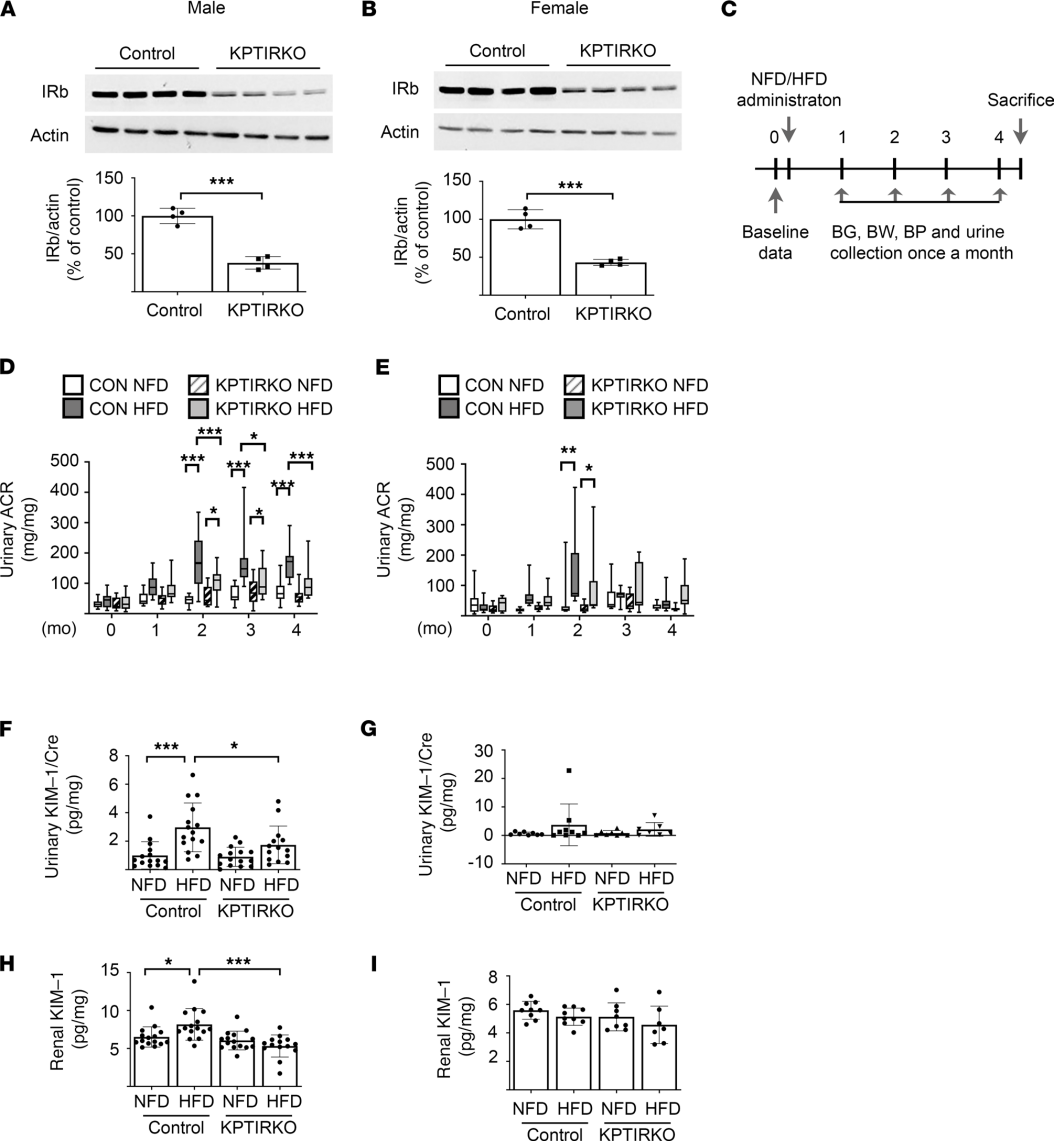
KPTIRKO mice are resistant to HFD-induced kidney injury. (**A** and **B**) Immunoblotting showed significantly reduced renal cortical IR expression in male and female mice, respectively (4 mice in each group, ***P < 0.001 by *t* test). (**C**) Schematic of administration of NFD or HFD and data collection. Blood glucose (BG), body weight (BW), blood pressure (BP) were measured and urine was collected for analysis once per month (14–15 male mice per group, 7–9 mice female mice per group). (**D** and **E**) HFD-induced albuminuria, measured as the urinary albumin-to-creatinine ratio (ACR), was inhibited in KPTIRKO male (*n =* 14–15 per group) and female (*n =* 7–9 per group) mice (*P < 0.05, **P < 0.01, ***P < 0.001 by 2-way ANOVA). (**F** and **G**) HFD increased the urinary KIM-1–to-creatinine ratio in male control mice but not in KPTIRKO mice (*n =* 14–15 per group) (**F**); it was unaffected in female mice (**G**) (*n =* 7–8 per group) (*P < 0.05, ***P < 0.001 by 1-way ANOVA and post hoc analysis using Tukey’s multiple-comparisons test). (**H** and **I**) HFD led to an increase in renal cortical KIM-1 expression in male control but not KPTIRKO mice (*n =* 14–15 per group); HFD did not affect KIM-1 expression in female mice of either genotype (*n =* 7–9 per group) (*P < 0.05, ***P < 0.001 by 1-way ANOVA and post hoc analysis using Tukey’s multiple-comparisons test).

Immunoblotting showed that IRβ chain expression was reduced by approximately 60% in the renal cortexes of both male and female KPTIRKO mice (Figure 1, A and B). The residual expression of IRβ in the renal cortex is probably due to unaltered expression in the glomeruli, ascending limb of loops of Henle, distal tubules, and collecting ducts. Additionally, because *Sglt2* is expressed mostly in the S1 segment of the proximal tubules (23), it is likely that IR is intact in the S2 and S3 segments (24). IRβ expression in KPTIRKO mice was unchanged in the liver, skeletal muscle, heart, brain, and pancreas (Supplemental Figure 2) as well as the lung and spleen (data not shown). There was no change in the IGF-I receptor (IGF-IR) expression in the kidney cortexes of male and female KPTIRKO mice (Supplemental Figure 3), indicating that there was no compensatory increase in IGF-IR expression for the loss of IRβ. Survey of signaling kinases showed no change in the phosphorylation of Akt or Erk in the renal cortexes of KPTIRKO mice compared with that in control mice (Supplemental Figure 3). There was no difference in the expression of renal cortical content of matrix proteins laminin or fibronectin in the KPTIRKO mice relative to that in control mice (data not shown). Serum chemistries showed no changes in blood urea nitrogen and serum creatinine (Supplemental Table 1).

### Effect of HFD on food consumption and body weight.

Control and KPTIRKO mice were randomized to receive normal-fat diet (NFD) or HFD for 4 months and underwent monthly evaluation, as shown in Figure 1C. There was no difference in body weight at baseline before randomization to NFD versus HFD among mice assigned to the 4 groups (control mice on NFD, control mice on HFD, KPTIRKO mice on NFD, and KPTIRKO mice on HFD). HFD resulted in weight gain to an equal extent in male control and KPTIRKO mice (Supplemental Figure 4). Similarly, female control and KPTIRKO mice also gained comparable weight on HFD (Supplemental Figure 4). Food consumption measurement showed that it was higher initially in HFD-fed control and KPTIRKO mice relative to NFD fed mice in both sexes; however, food consumption decreased in HFD-fed mice from that time point comparable to NFD fed mice, despite continued weight gain (Supplemental Figure 4).

### HFD-induced albuminuria and tubular injury are reduced in KPTIRKO mice.

There was no difference in the urinary albumin-to-creatinine ratio (ACR) between male and female control and KPTIRKO mice on NFD at baseline and for the duration of the study. In male control mice, HFD increased the urinary ACR at 2, 3, and 4 months. In KPTIRKO mice, HFD increased ACR only at 2 and 3 months, and from the second month, ACR was significantly lower in KPTIRKO mice relative to control mice until the end of the study (Figure 1D). Over the 4-month period of HFD, control mice had a significantly longer duration of albuminuria compared with HFD-fed KPTIRKO mice. In female mice, HFD increased ACR, which was significantly lower in the KPTIRKO mice, only at the second month time point (Figure 1E).

Renal cortical nephrin content was unchanged among the HFD-fed groups compared with NFD-fed groups, suggesting that albuminuria was likely not due to podocyte injury (Supplemental Figure 5). Increased urinary kidney injury molecule-1 (KIM-1) excretion is an index of proximal tubular injury (25). The ratio of urinary KIM-1 to creatinine and renal cortical KIM-1 content were increased in HFD-fed male control mice but not in KPTIRKO mice (Figure 1, F and H); these parameters did not change in HFD-fed female mice of both genotypes (Figure 1, G and I). These observations show the following: (a) HFD-induced albuminuria is dependent on proximal tubule IR in male and female mice. (b) HFD-induced albuminuria is likely due to proximal tubular injury. (c) HFD-induced albuminuria is milder in female mice, which resist HFD-induced proximal tubular injury.

### HFD-induced hypertension is ameliorated in male KPTIRKO mice.

**Figure 2:**
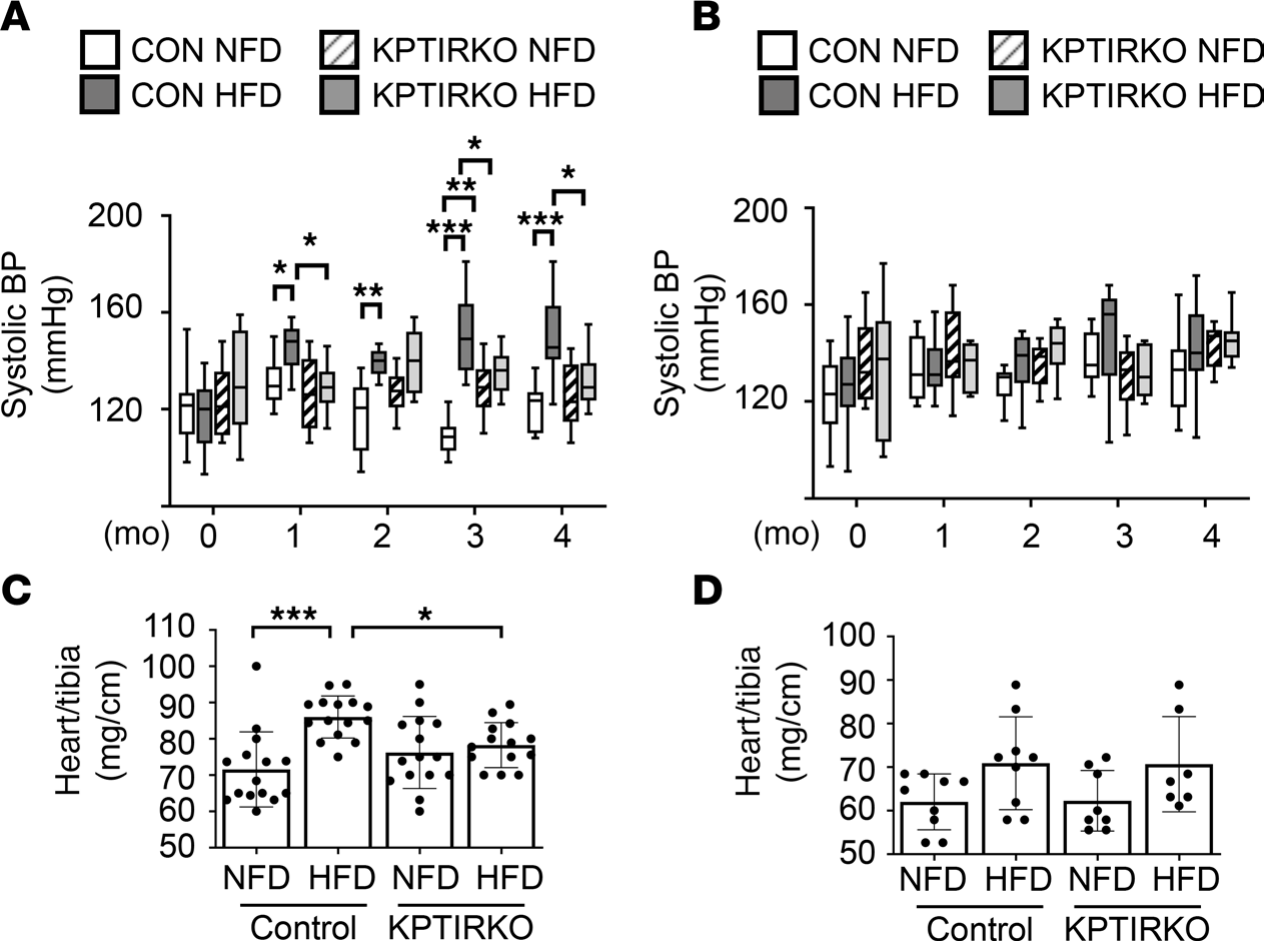
HFD-induced hypertension and heart hypertrophy are mitigated in KPTIRKO mice. (**A** and **B**) Systolic blood pressure (BP) was increased on HFD in male control mice but not KPTIRKO mice (*n =* 14–15 per group). It was unchanged by HFD in female control and KPTIRKO mice (*n =* 7–9 per group) (*P < 0.05, **P < 0.01, ***P < 0.001 by 2-way ANOVA and post hoc analysis using Tukey’s multiple-comparisons test). (**C** and **D**) HFD led to an increase in the heart weight–to-tibia ratio in male control mice but not in KPTIRKO mice (*n =* 14–15 per group); the ratio was unchanged in female mice on HFD (*n =* 7–9 per group) (*P < 0.05, ***P < 0.001 by 1-way ANOVA and post hoc analysis using Tukey’s multiple-comparisons test). In the graphs, each dot represents 1 mouse. Data are presented as mean ± SD.

HFD was associated with a sustained increase in systolic blood pressure in male control mice for the duration of the study. Male KPTIRKO mice demonstrated a lower systolic blood pressure at nearly all time points (Figure 2A). There was no significant difference between systolic blood pressures of male control and KPTIRKO mice on NFD at most time points. The heart-to-tibia ratio was increased in HFD-fed male control mice with hypertension consistent with cardiac hypertrophy; this did not occur in HFD-fed male KPTIRKO mice, suggesting absence of cardiac hypertrophy, possibly related to lack of hypertension (Figure 2C). These data agree with those in the report by Wang et al. (26). Systolic blood pressure generally did not exceed 140 mm Hg on either diet in female mice of either genotype (Figure 2B); correspondingly, there was no change in the heart-to-tibia ratio in female mice fed HFD (Figure 2D). Thus, male but not female mice are susceptible to HFD-induced hypertension and heart hypertrophy, which requires the presence of IR in the proximal tubule.

### HFD-induced renal matrix changes are reduced in male KPTIRKO mice.

**Figure 3:**
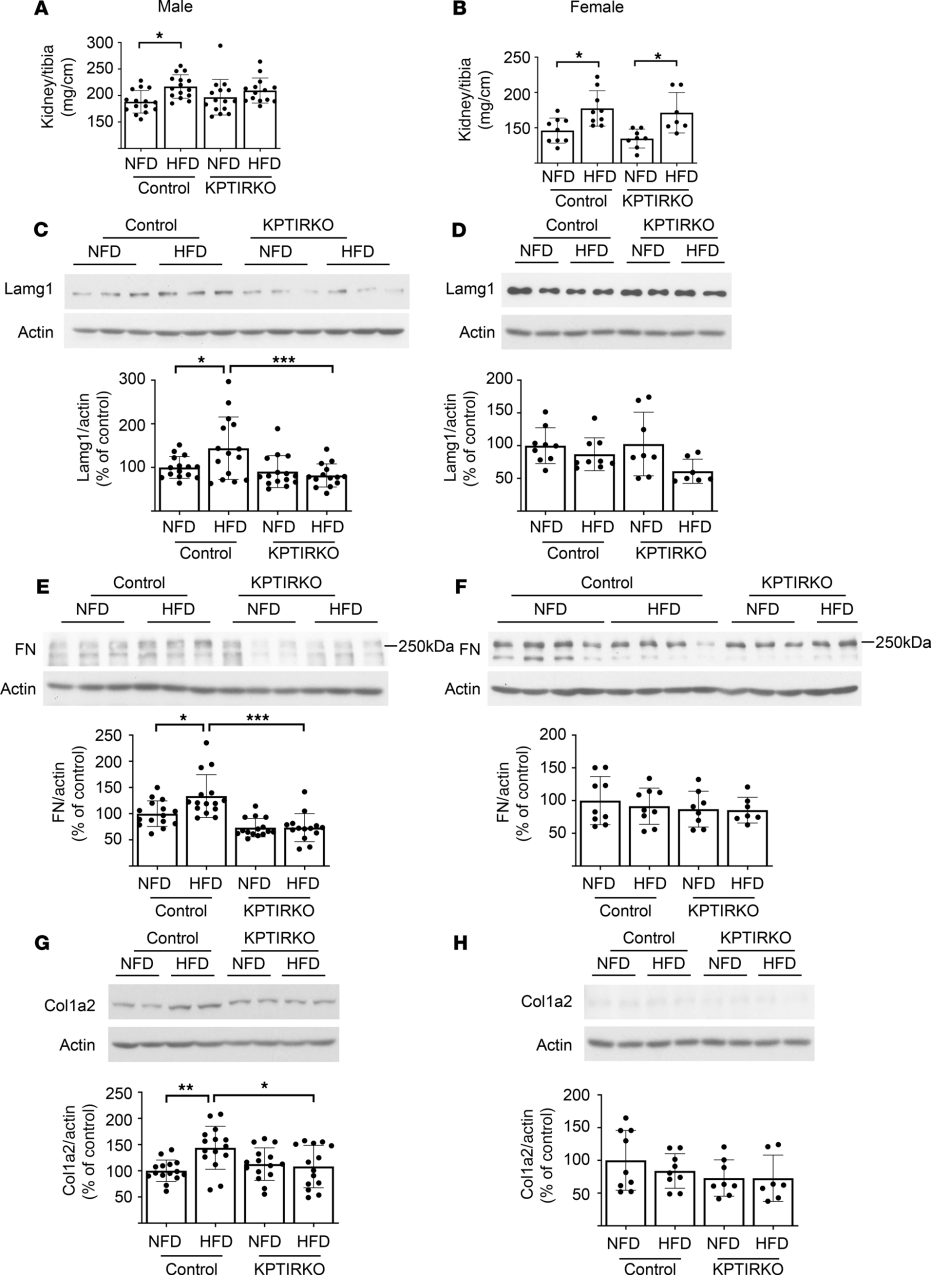
KPTIRKO mice are protected from matrix increase following HFD. (**A** and **B**) HFD increased the kidney-to-tibia ratio, indicating hypertrophy in male control mice but not in KPTIRKO mice (*n =* 14–15 per group). HFD increased the kidney-to-tibia ratio in female mice of both genotypes (*n =* 7–9 per group). (**C**–**H**) Immunoblotting of renal cortical extracts showed increase in the expression of laminin γ1, fibronectin, and collagen 1α2 in male control but not in KPTIRKO mice (**C**, **E**, and **G**) (*n =* 14–15 per group); HFD did not affect the renal cortical content of these proteins in female mice of either genotype (**D**, **F**, and **H**) (*n =* 7–9 per group) (*P < 0.05, **P < 0.01, ***P < 0.001 by 1-way ANOVA and post hoc analysis using Tukey’s multiple-comparisons test). In the graphs, each dot represents 1 mouse. Data are presented as mean ± SD.

The kidney-to-tibia ratio was increased in male control mice but not in male KPTIRKO mice fed HFD (Figure 3A), suggesting that IR is required for kidney hypertrophy to occur. Interestingly, HFD induced kidney hypertrophy in both genotypes in female mice (Figure 3B), suggesting that either IR in other tubular segments and/or other growth regulators may be sufficient to induce HFD-induced kidney hypertrophy. We examined the levels of matrix proteins that contribute to renal fibrosis. Immunoblotting showed increased renal cortical content of laminin γ1, fibronectin, and collagen I α2 in male control mice fed HFD but not in male KPTIRKO mice (Figure 3, C, E, and G). There was no change in the expression of these matrix proteins in female mice fed HFD (Figure 3, D, F, and H). These data demonstrate that (a) in male mice proximal tubular IR is required for kidney hypertrophy and increase in renal cortical matrix proteins following HFD and (b) that female mice resist HFD-induced matrix protein increase in the renal cortex, although they manifest renal hypertrophy.

### HFD-induced signaling pathways are unchanged in male KPTIRKO mice.

**Figure 4:**
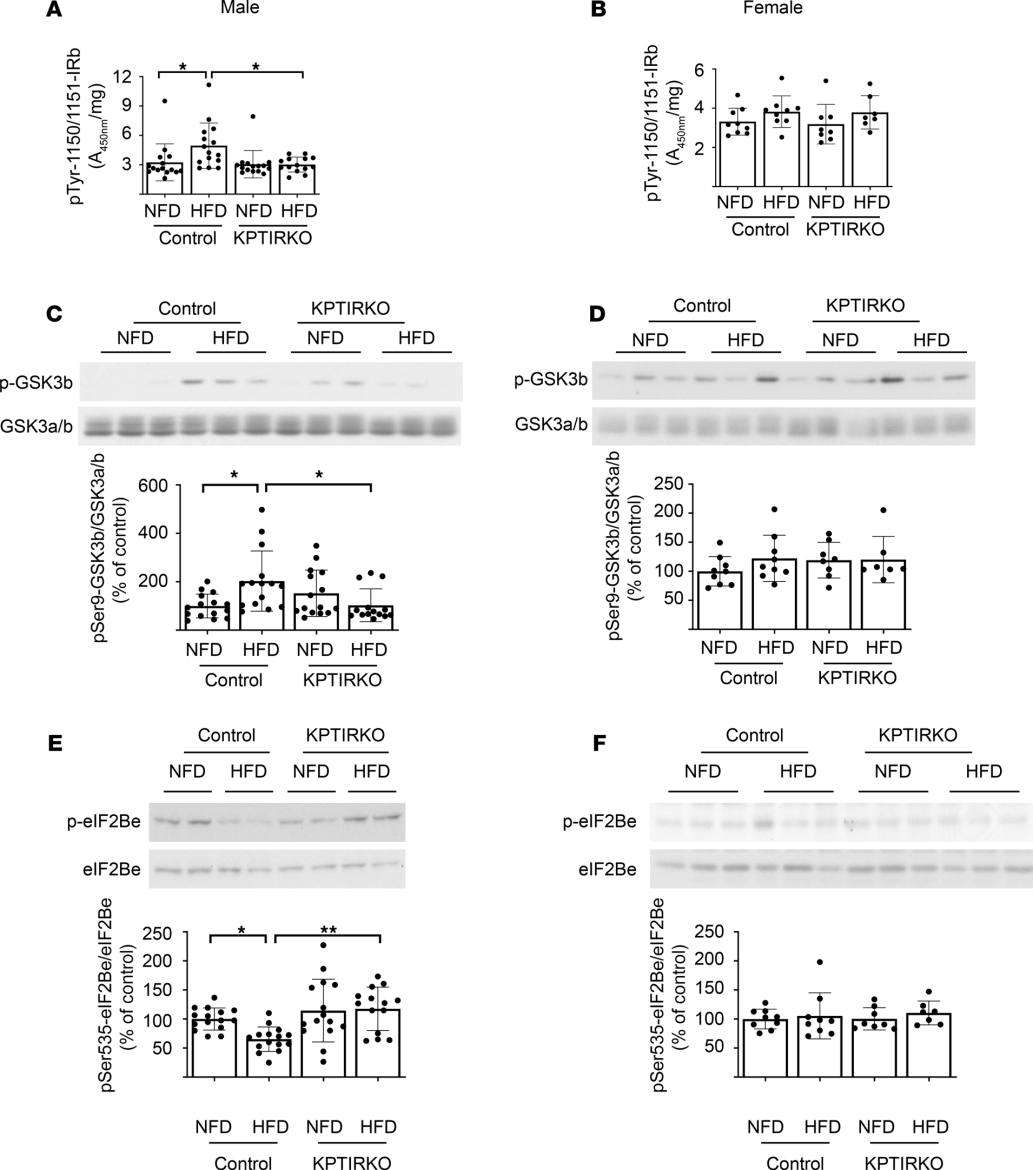
Signaling studies-1. (**A** and **B**) ELISA was performed to assess IR Tyr-1150/1151 phosphorylation. HFD increased this parameter in the renal cortexes of male control mice but not in KPTIRKO mice (*n =* 14–15 per group). HFD did not affect it in female mice of either genotype (*n =* 7–9 per group). (**C** and **D**) HFD increased renal cortical Ser-9 phosphorylation of GSK3β, indicating Akt activation in male control but not KPTIRKO mice (*n =* 14–15 per group); it was unchanged in female mice (*n =* 7–9 per group). (**E** and **F**) Kidney cortical phosphorylation of eIF2Bε, an index of GSK3β activity, was decreased in HFD-fed male control mice but not in KPTIRKO mice (*n =* 14–15 per group); it remained unaltered in female control and KPTIRKO mice (*n =* 7–9 per group) (*P < 0.05, **P < 0.01, by 1-way ANOVA and post hoc analysis using Tukey’s multiple-comparisons test).

Insulin is a powerful inducer of protein synthesis via regulation at the level of mRNA translation in kidney cells (19, 21). Hyperinsulinemia could stimulate the synthesis of matrix proteins in HFD-induced kidney injury. We examined signaling events related to IR activation that are linked to protein synthesis. IR signaling begins with phosphorylation at key tyrosine residues 1150 and 1151 in the IRβ chain (27). Renal cortical IR tyrosine phosphorylation at Tyr1150/1151 was increased in HFD-fed male control mice but not in KPTIRKO mice (Figure 4A). HFD did not result in changes in IR Tyr-1150/1151 phosphorylation in female mice of either genotype (Figure 4B). On NFD, fasting serum insulin levels were similar in male control and KPTIRKO mice; HFD resulted in hyperinsulinemia in both control and KPTIRKO mice to the same extent (Supplemental Figure 6). There was no difference in random serum insulin levels among the 4 groups of female mice (Supplemental Figure 6). IR activation leads to activation of Akt, which controls downstream signaling events leading to protein synthesis (28, 29). We assessed Akt activity by examining the phosphorylation of glycogen synthase kinase 3β (GSK3β), a direct substrate of Akt. Phosphorylation of GSK3β by Akt at Ser9 leads to inhibition of its activity and removes inhibition on protein synthesis (30, 31), including in kidney cells (32). Conversely, stimulation of GSK3β inhibits synthesis of proteins in the kidney (33). HFD increased the renal cortical GSK3β phosphorylation at Ser9 in male control mice but not in KPTIRKO mice (Figure 4C). HFD did not affect GSK3β phosphorylation in female control or KPTIRKO mice (Figure 4D). Akt-induced inhibition of GSK3β reduces Ser535 phosphorylation of its direct substrate, eukaryotic initiation factor 2B (eIF2Bε, refs. 32, 34). eIF2B is a heteropentamer and its ε component promotes GDP/GTP exchange on eIF2, a key regulatory step in the initiation phase of mRNA translation (35). HFD reduced eIF2Bε phosphorylation in the renal cortexes of male control mice consistent with decreased GSK3β activity but not in HFD-fed KPTIRKO mice (Figure 4E). There was no change in eIF2Bε phosphorylation in female mice on HFD (Figure 4F), in alignment with unchanged activity of GSK3β.

**Figure 5:**
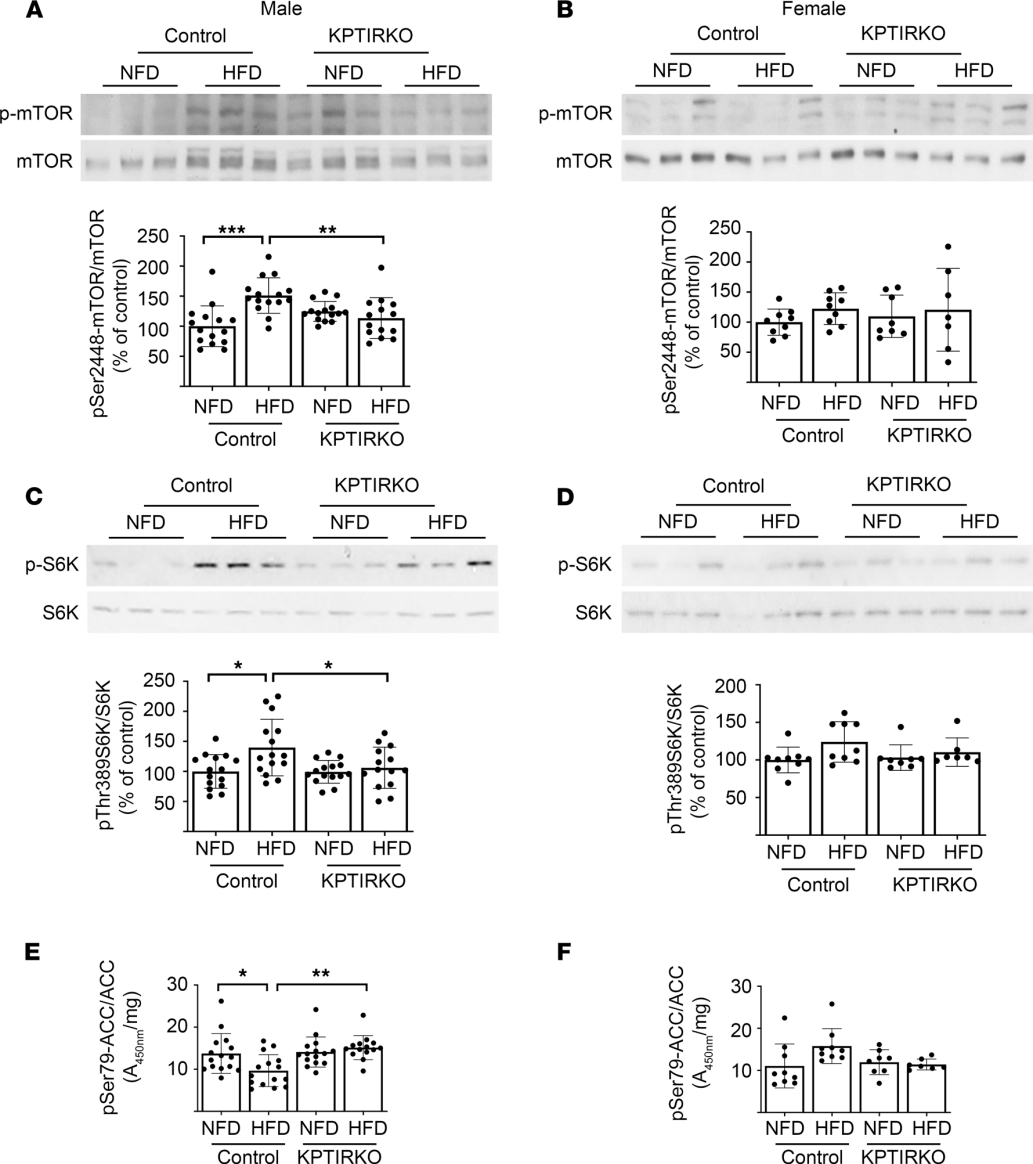
Signaling studies-2. (**A**–**D**) Immunoblotting showed that Ser-2448 phosphorylation of mTOR and Thr-389 phosphorylation of p70S6 kinase were increased, indicating mTORC1 activation in the renal cortexes of HFD-fed male control but not KPTIRKO mice (*n =* 14–15 per group). HFD did not affect these parameters in female mice (*n =* 7–9 per group). (**E** and **F**) Ser-79 phosphorylation of ACC was measured by ELISA as an index of AMPK activity. HFD inhibited AMPK in male control mice but not in KPTIRKO mice (*n =* 14–15 per group); it was unchanged in female mice of both genotypes (*n =* 7–9 per group) (*P < 0.05, **P < 0.01, ***P < 0.001 by 1-way ANOVA and post hoc analysis using Tukey’s multiple-comparisons test). In the graphs, each dot represents 1 mouse. Data are presented as mean ± SD.

Downstream of Akt, mTORC1 governs mRNA translation by directly phosphorylating p70S6 kinase and eukaryotic initiation factor 4E binding protein (29). Akt activates mTORC1 in several ways. It phosphorylates tuberous sclerosis 1 (TSC-1) and AMPK, leading to activation of Rheb, which promotes mTORC1 activity (29). Additionally, it phosphorylates PRAS40, which is a tonic inhibitor of mTORC1. Activation of mTORC1 involves Ser2448 phosphorylation. HFD promoted mTOR phosphorylation at this site in male control mice but not in male KPTIRKO mice (Figure 5A). HFD did not affect mTORC1 phosphorylation in female mice of either genotype (Figure 5B). Renal cortical p70S6 kinase phosphorylation was increased in male mice fed HFD but not in KPTIRKO mice on the same diet (Figure 5C); it was unchanged among the 4 groups of female mice (Figure 5D). Activation of mTORC1-p70S6 kinase regulates mRNA translation, a rate-limiting step in protein synthesis (29, 36), and is likely to be related to increase in synthesis of proteins, including matrix proteins, in HFD-fed mice.

The energy sensor, AMPK, is a major regulator of protein synthesis in the kidney; its activity is inhibited in renal injury associated with diabetes (10, 37, 38). AMPK activity is also inhibited in HFD-associated kidney injury (9, 39). Akt inhibits AMPK by phosphorylation (40). We examined AMPK activity by studying Ser79 phosphorylation of acetyl-CoA carboxylase (ACC), its direct substrate. HFD inhibited ACC phosphorylation in the renal cortexes of male control mice (Figure 5E). It was unchanged in HFD-fed male KPTIRKO mice compared with NFD fed mice, and, it was significantly higher compared with HFD-fed control mice (Figure 5E). In female mice, ACC phosphorylation showed a trend toward increase in HFD-fed controls whereas it was unchanged in KPTIRKO mice (Figure 5F). These data collectively show the following: (a) HFD induces renal cortical IR activation in male mice, leading to stimulation of Akt and inhibition of AMPK, and, increase in the activity of mTORC1, which governs mRNA translation. We have previously shown that these events facilitate synthesis of proteins, including matrix proteins (20, 32, 33). (b) In male mice, IR is required for stimulation of Akt, mRNA translation signaling events, and AMPK inactivation following HFD. (c) HFD does not promote IR phosphorylation and downstream signaling events in female mice. The trend toward increased AMPK activity in female control HFD may represent a defense against stimulation of protein synthesis events by HFD.

### HFD reduces hydrogen sulfide generation only in male control but not KPTIRKO mice.

**Figure 6:**
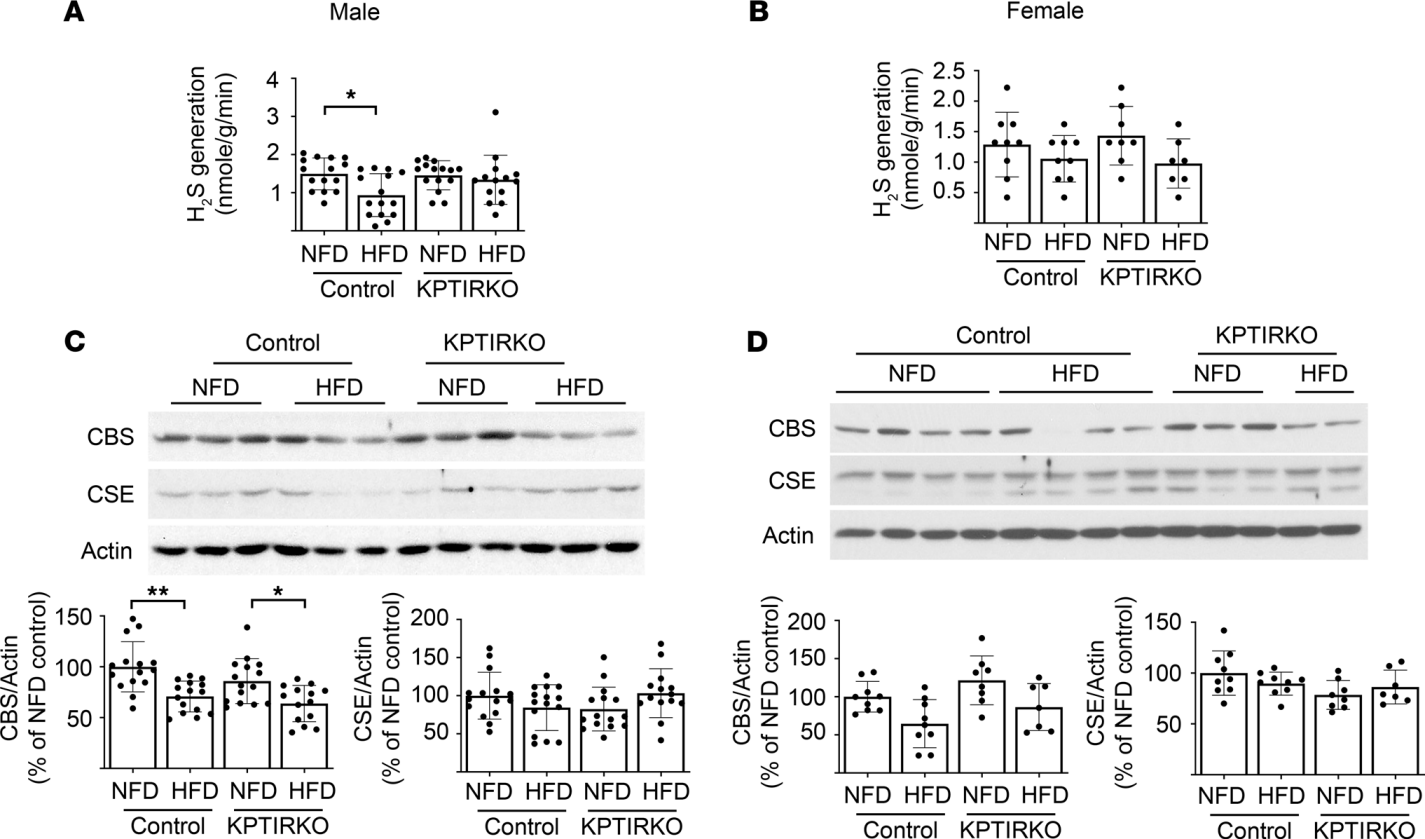
HFD regulation of hydrogen sulfide in the kidney. (**A** and **B**) HFD inhibited renal cortical hydrogen sulfide (H_2_S) generation in male control but not KPTIRKO mice (*n =* 14–15 per group); HFD did not change H_2_S generation in female control or KPTIRKO mice (*n =* 7–9 per group). (**C** and **D**) Immunoblotting showed that HFD reduced the expression of cystathionine β-synthase (CBS) but not cystathionine γ-lyase (CSE) in male control and KPTIRKO mice (*n =* 14–15 per group). HFD did not affect the expression of these enzymes in female mice of either genotype (*n =* 7–9 per group) (*P < 0.05, **P < 0.01 by 1-way ANOVA and post hoc analysis using Tukey’s multiple-comparisons test). In the graphs, each dot represents 1 mouse. Data are presented as mean ± SD.

Hydrogen sulfide (H_2_S), a gasotransmitter, is constitutionally synthesized by the kidney. It regulates glomerular filtration rate, renal blood flow, and tubular handling of electrolytes (41). H_2_S is generated in the kidney in cytosolic and mitochondrial metabolic reactions related to L-cysteine, with the participation of cystathionine β-synthase (CBS), cystathionine γ-lyase (CSE), and 3-mercaptopyruvate sulfotransferase (42, 43). An additional pathway involving D-cysteine also operates in the kidney (44). We have previously reported that CBS and CSE expression, and generation of H_2_S, are reduced in the renal cortexes of aged mice, marmosets, and mice with type 1 and type 2 diabetes (45–47). Aging-associated albuminuria, renal fibrosis, and activation of the IR signaling pathway are also associated with H_2_S deficiency in mice (45). These injury parameters in aged mice are ameliorated by the administration of sodium hydrosulfide (NaHS), a source of H_2_S (45). We examined the status of H_2_S in HFD-fed mice. H_2_S generation by the kidney cortexes was reduced in HFD-fed male control mice but not in KPTIRKO mice (Figure 6A). In female mice, HFD did not affect H_2_S generation following HFD in either genotype (Figure 6B). HFD reduced the expression of CBS but not CSE in both male control and KPTIRKO mice (Figure 6C). In female mice, CBS expression showed a trend toward reduction in HFD-fed control mice but not in KPTIRKO mice; CSE content was unaffected (Figure 6D). These data suggest the following. (a) In male mice, IR is involved in reducing H_2_S generation in HFD-fed mice. (b) Maintenance of H_2_S generation in HFD-fed KPTIRKO mice, despite reduced CBS expression, suggests that CBS-independent mechanisms maintain H_2_S production. (c) In female mice, H_2_S generation is unchanged following HFD in control mice although CBS expression tended to decrease, indicating existence of compensatory mechanisms to maintain H_2_S generation. Intact renal cortical H_2_S production may aid the female mice in resisting HFD-induced kidney injury.

### Metabolic studies.

**Figure 7:**
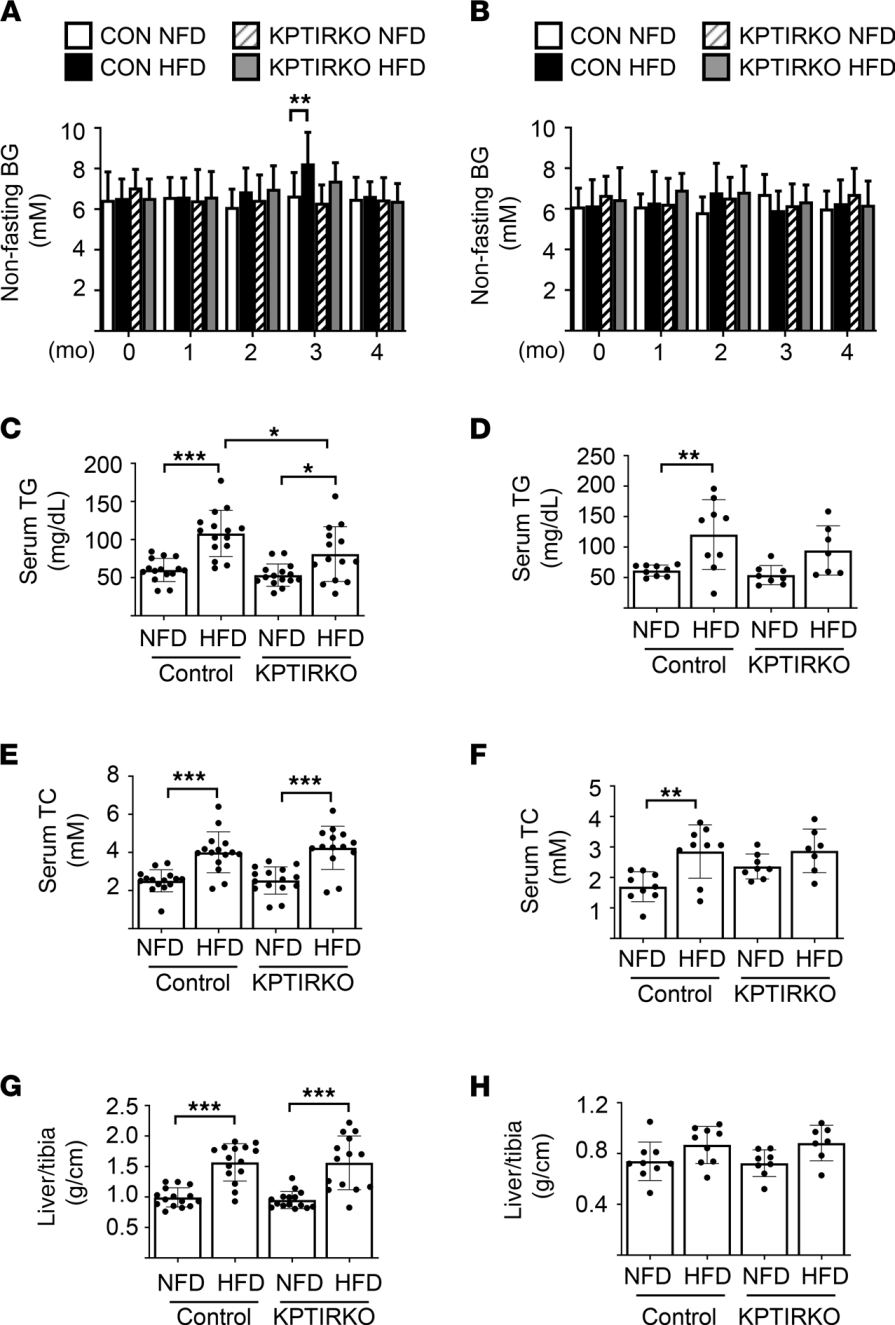
HFD regulation of metabolic parameters. (**A** and **B**) HFD did not affect nonfasting blood glucose in male (*n =* 14–15 per group) or female mice of either genotype (*n =* 7–9 per group), except once at 3 months in male control mice. (**P < 0.01 by 2-way ANOVA and post hoc analysis using Tukey’s multiple-comparisons test). (**C** and **D**) Serum triglyceride (TG) level was increased by HFD in male control mice and increased to a lesser extent in KPTIRKO mice (*n =* 14–15 per group). It was also increased by HFD in female control mice but not in KPTIRKO mice (*n =* 7–9 per group). (**E** and **F**) HFD increased serum total cholesterol (TC) level in male control and KPTIRKO mice (*n =* 14–15 per group). The parameter was increased by HFD in female control mice; the change was not significant in female KPTIRKO mice (*n =* 7–9 per group). (**G** and **H**) The liver-to-tibia ratio was increased by HFD in male control and KPTIRKO mice (*n =* 14–15 per group); HFD did not change the ratio in female mice of either genotype (*n =* 7–9 per group). (*P < 0.05, **P < 0.01, ***P < 0.001 by 1-way ANOVA and post hoc analysis using Tukey’s multiple-comparisons test). In the graphs, each dot represents 1 mouse. Data are presented as mean ± SD.

Random blood glucose concentration was measured once per month. In male control and KPTIRKO mice on NFD or HFD, blood glucose stayed below 8 mmol/L, except for 1 spike to 8 mmol/L at month 3 in HFD-fed control mice (Figure 7A). In female mice, blood glucose remained below 8 mmol/L in both genotypes (Figure 7B). HFD increased serum triglycerides in male and female control mice; the increase was attenuated in male KPTIRKO mice with a similar trend in female KPTIRKO mice (Figure 7, C and D). HFD increased serum cholesterol in male control and KPTIRKO mice (Figure 7E). HFD increased serum cholesterol in HFD-fed female control mice but not in KPTIRKO mice (Figure 7F). The liver-to-tibia ratio was increased in male control and KPTIRKO mice fed HFD, indicating a similar hepatic response to HFD in both genotypes (Figure 7G). This parameter was unchanged in female mice regardless of the genotype (Figure 7H). These data indicate the following. (a) Serum triglyceride elevation due to HFD is lower in KPTIRKO mice compared with control mice, suggesting a possible regulatory role for proximal tubule IR in systemic triglyceride metabolism. (b) Female mice resist hepatic enlargement in HFD. Taken together our data show that the amelioration of HFD-induced kidney injury in male KPTIRKO mice is due to the absence of IR in the proximal tubules.

## Discussion

Our data demonstrate that proximal tubular epithelial cell IR activation by hyperinsulinemia in HFD-fed mice is required for the development of albuminuria, hypertension, matrix protein accumulation, renal hypertrophy, dyslipidemia, and stimulation of signaling pathways involved in protein synthesis. In states of insulin resistance, the kidney may be an “innocent bystander,” subject to the injurious effects of hyperinsulinemia. The increase in circulating insulin could be due to several mechanisms: (a) HFD-induced insulin resistance in extrarenal tissues; (b) direct nutrient stimulation of β cells of the islets secondary to increased caloric intake (48, 49); (c) stimulation of insulin secretion by the gut-derived incretin hormones; (5) decreased metabolic clearance rate of insulin by the liver due to hepatic insulin resistance; (6) reduced renal insulin degradation due to proximal tubular IR knockout; and (7) a combination of the above.

In the kidney, IR is expressed by the constituent cells of the glomerulus and all the segments of the tubules (50, 51). IR knockout in specific parts of the nephron have revealed a segment- and cell-specific role of insulin in regulating physiologic functions. IR contributes to normal nephron function by preventing loss of albumin during glomerular filtration, proximal tubular maintenance of glucose homeostasis, sodium reabsorption, and defense against urinary tract infection (52–55); these effects of insulin on the kidney are achieved by selective recruitment of IR substrate 1 (IRS-1) versus IRS-2 (56). Podocyte IR-knockout mice manifest albuminuria, thickening of the glomerular basement membrane, and glomerulosclerosis mimicking diabetic injury in the presence of euglycemia (52). Under physiologic conditions, the minute amounts of albumin that pass the glomerular filtration barrier are reabsorbed by the proximal tubules mainly by the megalin-cubilin system, which is stimulated by insulin (57). Thus, absence of IR in proximal tubules should lead to albuminuria. However, in our study the basal urinary albumin excretion in KPTIRKO mice was similar that of control mice on NFD, indicating that IR-independent mechanisms compensate to conserve albumin endocytosis. Albuminuria seen in HFD-fed control mice was absent in KPTIRKO mice, suggesting that the proximal tubule IR mediates obesity-induced urinary protein losses. Normal nephrin expression and an increase in urinary and renal cortical KIM-1, an index of proximal tubular injury, further support the notion that HFD-induced albuminuria in control mice is due to injury to the proximal tubules rather than podocytes; absence of changes in KIM-1 in KPTIRKO mice on HFD indicates that IR mediates proximal tubular injury.

The kidney is an important site of glucose production by the process of gluconeogenesis and contributes to about 20% of endogenous glucose production under postabsorptive conditions (58). Hyperglycemia has been reported in Ggt-Cre–mediated proximal tubular IR-knockout mice due to stimulation of gluconeogenesis (53). In our study, blood glucose levels monitored every month did not reveal sustained hyperglycemia in NFD-fed KPTIRKO mice; further, even the mild elevation in blood glucose seen in HFD-fed control and KPTIRKO mice was within the normal range. Possible explanations for the difference between the 2 studies could be as follows. (a) In our study, IR was absent only in the S1 segment of the proximal tubule, whereas the IR deletion may have been more extensive in the study by Tiwari et al. (53). (b) Differences in the genetic background of mice may have played a role. Tiwari et al. used 129/Sv/C57BL6 mice whereas C57BL6 mice were employed in our study (53).

Since the pioneering studies of DeFronzo et al. on the effects of insulin on renal handling of sodium (55), many groups have used targeted deletion of IR in tubular segments to elucidate its role in hypertension. Kap-Cre–mediated deletion of IR, mainly involving the thick ascending limb and the collecting duct, led to an increase in systolic and diastolic blood pressures (59). Since IR was intact in the proximal tubules in this study, it is possible that it contributed to hypertension by increasing sodium reabsorption. In our study, HFD increased blood pressure in control mice but not in KPTIRKO mice, suggesting that proximal tubular IR was involved in the genesis of hypertension. Nakamura et al. have shown that insulin recruits the IRS2/Akt2/mTORC2 signaling pathway to activate the sodium bicarbonate cotransporter to promote sodium reabsorption in the proximal tubule and contribute to hypertension in metabolic syndrome (60, 61). IR/mTORC2 axis is also involved in the stimulation of sodium reabsorption by activation of the sodium-chloride cotransporter in the distal convoluted tubule and the epithelial sodium channel in the collecting duct, which could also contribute to hypertension (62–64). While hypertension could have mediated some aspects of HFD-induced kidney injury, our data show that genesis of hypertension requires intact IR signaling.

In hyperinsulinemic states, such as obesity, not all tissues and not all pathways are insulin resistant (65). The term insulin sensitivity and its reciprocal, resistance, generally have been employed in the context of glucose uptake by cells. However, in insulin-resistant states, events other than glucose transport, e.g., protein synthesis, may be activated in insulin-responsive tissues, such as the kidney and the vessel wall. There are examples of IR-mediated injury in other tissues. In diabetes, retinal neovascularization was reduced in vascular endothelial specific IR-knockout mice; IGF-1 receptor activation had a less important role (66, 67). Hyperinsulinemia is an independent risk factor for coronary artery disease (68–70). Cardiac IR signaling is stimulated in a pressure overload model of heart failure, whereas selective cardiac deletion of IR ameliorates cardiac injury, demonstrating a direct role for IR in heart injury (26, 71). Of relevance to atherosclerosis, in insulin-resistant states, insulin’s ability to activate the IR signaling pathway in vascular smooth muscle cells is impaired, leading to decreased nitric oxide generation and accelerated atherogenesis (70). Consistent with these observations, insulin augments endothelial expression of endothelin-1 (72, 73), PAI-1 (74), and vascular smooth muscle cell proliferation (75). Insulin has been shown to activate the MAP kinase pathway, despite the presence of insulin resistance in the Akt pathway, leading to oxidative stress in the wall of blood vessels of human subjects with coronary atherosclerosis (76). It is likely that hyperinsulinemia, when it occurs in the setting of other metabolic syndrome components, is especially injurious to the arterial wall (77). IGF-1 can signal through IGF-1R, IR, or IGF-1R+IR hybrid receptors; however, IGF-1 is unlikely to be involved in our model because KPTIRKO mice resisted HFD-induced kidney injury.

Our data suggest a potentially novel mechanism of IR-mediated kidney injury in obesity. Decreased renal cortical production of H_2_S was seen in HFD-fed control mice but not in KPTIRKO mice, suggesting that IR activation leads to H_2_S deficiency. Recent studies have unraveled an important role for H_2_S in defending kidney integrity in a variety of models of acute kidney injury and CKD (43). In aging-associated kidney injury, renal cortical IR activation with stimulation of the Akt/mTORC1 pathway is associated with H_2_S deficiency (45). Administration of H_2_S improves IR activation and the aforementioned indices of kidney injury (45). In the aging kidney, H_2_S deficiency leads to IR activation, placing H_2_S upstream of IR. Reduction in renal cortical expression of H_2_S-generating enzymes is also seen in rodent models of type 1 and 2 diabetes (47).

IR mediation of HFD-associated kidney injury is sex dependent. Compared with males, the female KPTIRKO mice had the same extent of IR deletion in the renal cortex, and, on HFD, gained comparable body weight and developed hypercholesterolemia. However, they sustained a milder form of HFD-induced kidney injury, as indicated by less albuminuria. On HFD, the female KPTIRKO mice did not develop elevated blood pressure and consequent heart hypertrophy; they did not show matrix protein accumulation or stimulation of signaling reactions related to protein synthesis. Renal cortical and urinary KIM-1 contents were unaffected in female mice on HFD. The ability of female mice to defend against HFD-induced kidney injury may be related to maintaining AMPK activity and H_2_S generation. Impaired AMPK activity leads to kidney injury in diabetes and obesity, and conversely, stimulation of AMPK ameliorates kidney injury in those states (9, 37, 78). Deficiency in H_2_S generation is associated with kidney injury in diabetes and aging (45, 47, 79), while administration of H_2_S as NaHS ameliorates aging-associated kidney injury by stimulating AMPK activity and inhibiting IR activation associated Akt signaling related to protein synthesis (45). A similar salutary effect of H_2_S has also been reported in rodents with diabetic kidney injury (80). Female mice maintained normal H_2_S synthesis, despite reduction in CBS expression, indicating that compensation occurs at other sites in its synthetic pathway. Additionally, translating ribosome affinity purification analysis of proximal tubules has shown major differences in gene expression between male and female mice at baseline and following injury, suggesting a genetic mechanism for differential susceptibility (81). Many studies have shown that female sex is associated with lower incidence of hypertension, diabetic microangiopathy, progressive CKD, and death in CKD (82–85). Studies on mechanisms of sex differences in kidney injury suggest an injurious role for testosterone in males and a protective effect of estrogen in females (86–89).

A fascinating observation in the current study is that absence of IR in the proximal tubules reduces the extent of hypertriglyceridemia in HFD-induced obesity. This suggests a potential regulatory role for proximal tubular IR in the genesis of elevated triglycerides. More work is needed to understand if an axis exists between the kidney and other organs affecting uptake, synthesis, or degradation of triglycerides.

In conclusion, our data demonstrate a potentially new paradigm of kidney injury in HFD-induced obesity. Further exploration on renal IR-regulated cellular mechanisms involved in HFD-induced kidney injury is needed.

## Methods

### Materials and sources

We employed antibodies against the following: IRβ (catalog 3025, Cell Signaling Technology), Laminin γ1 (catalog sc-5584, Santa Cruz Biotechnology), fibronectin (catalog ab2413, Abcam), type I collagen α2 (catalog 14695-1-AP, Proteintech), phospho-Ser9-GSK3β (catalog 9323, Cell Signaling Technology), GSK3α/β (catalog sc-56913, Santa Cruz Biotechnology), phospho-Ser539-eIF2Bε (catalog 44-530G, Thermo Fisher Scientific), eIF2Bε (catalog 3595, Cell Signaling Technology), phospho-Ser2448-mTOR (catalog 2971, Cell Signaling Technology), mTOR (catalog 2972, Cell Signaling Technology), phospho-Thr389-p70S6 kinase (catalog 9205, Cell Signaling Technology), p70S6 kinase (catalog 9202, Cell Signaling Technology), CBS (catalog sc-67154, Santa Cruz Biotechnology), CSE (catalog sc-135203, Santa Cruz Biotechnology), IGF-1 receptor (catalog 3027, Cell Signaling Technology), phospho-Thr202/Tyr204-Erk (catalog 4377, Cell Signaling Technology), Erk (catalog 9102, Cell Signaling Technology), phospho-Ser473-Akt (catalog 9271, Cell Signaling Technology), Akt (catalog 9272, Cell Signaling Technology), nephrin (catalog ab58968, Abcam), and actin (catalog A2066, MilliporeSigma). Commercial ELISA kits were used for measuring phospho-Tyr-1150/1151-IR (catalog 7082, Cell Signaling Technology) and phospho-Ser79-ACC (catalog 7986, Cell Signaling Technology).

### Animals

#### Generation of KPTIRKO mice.

Sodium-dependent glucose transporter 2 (Sglt2) is located predominantly in the S1 segment of the proximal tubules (23) and governs glucose reabsorption; thus, the *Sglt2* promoter is specific for proximal tubule targeting (90). We confirmed the specificity of Sglt2 for the proximal tubules by crossing *Sglt2 Cre* mice with Rosa26 reporter mice designed to express LacZ; in the hybrid mice, β-galactosidase activity detected by X-gal staining was seen only in the proximal tubules of the kidney and not in the other parts of nephron or other organs (Supplemental Figure 7). We generated KPTIRKO mice by crossing *Sglt2 Cre* mice (provided by I. Rubera and M. Tauc, LP2M CNRS/Université Côte d’Azur laboratory, Nice, France; the strain is now archived and distributed by the European Mouse Mutant Archive, https://www.infrafrontier.eu/resources-and-services/access-emma-mouse-resources_ with *Ir-loxp* mice, in which exon 4 of the *Irβ* chain is floxed (provided in-house; catalog 006955, The Jackson Laboratory). *Ir-loxp Cre*–negative littermates were used as the control mice (Supplemental Figure 7). Initially, 8 generations of back crossing were done to bring *Ir-Loxp* mice to the C57BL6 genotype. At the beginning of Cre-Lox crossing both mice were on C57BL6 background.

We used a model of HFD-induced obesity in which hyperinsulinemia occurs in association with normal blood glucose levels. We randomized 5- to 8-month-old male and female control and KPTIRKO mice to receive HFD or NFD for 4 months. In the HFD, 60% of the calories are derived from fat (D12492, Research Diets Inc.), whereas 10% of the calories are fat derived in the NFD (D12450J, Research Diets). Three separate batches (male) and 2 separate batches (female) of mice were studied (male: control NFD, *n =* 15; control HFD, *n =* 15; KPTIRKO NFD, *n =* 15; KPTIRKO HFD, *n =* 14; and female: control NFD, *n =* 9; control HFD, *n =* 9; KPTIRKO NFD, *n =* 8; KPTIRKO HFD, *n =* 7).

#### Genotyping.

Lysis buffer (50 mM KCl, 10 mM Tris-HCl pH 8.3, 2.5 mM MgCl_2_, 0.1 mg/mL gelatin, 0.45% IGEPAL-CA630, 0.45% Tween 20, and 60 μg/mL Proteinase K (Thermo Fisher Scientific) was used to prepare DNA from the tails of mice. *Cre*-recombinase gene was amplified using the following primers: forward, 5′-AGGTTCGTTCACTCATGGA-3′, and reverse, 5′-TCGACCAGTTTAGTTACCC-3′. We used the following primers (Jackson Laboratory genotyping protocol) to amplify the region in the targeted *Ir* allele spanning the loxp sites: forward, 5′-GGGGCAGTGAGTATTTTGGA-3′, and reverse, 5′-TGGCCGTGAAAGTTAAGAGG-3′. The band size of the mutant allele was 145 bp, and the band size of the wild-type recombined allele had a size of 105 bp (Supplemental Figure 7).

#### BP measurement.

The tail cuff method was employed to measure blood pressure as previously described (22).

#### Immunoblotting.

SDS PAGE analysis of proteins and protocols for incubation with respective antibodies have been described previously (45, 47, 79, 91).

#### Urinary ACR ratio and blood glucose measurement.

We employed analytical kits to measure albumin (catalog E101 and E90-134, Bethyl Laboratories Inc.) and creatinine (catalog ADI-907-030A, Enzo Life Sciences Inc.). Blood glucose concentration was measured by a glucometer (Ascensia Diabetes Care US Inc.).

#### IR tyrosine phosphorylation and phosphorylation of ACC.

IR tyrosine phosphorylation and phosphorylation of ACC were measured by using ELISA kits according to the instructions provided by the manufacturers.

#### Insulin measurement.

Mouse serum insulin levels were determined using the mouse metabolic hormone panel according to the manufacturer’s instructions (Milliplex MMHMAG-44K, MilliporeSigma). Assays were performed on a MAGPIX Multiplexing instrument, and data were analyzed using xPONENT 4.2 software.

### Statistics

Data were expressed as mean ± SD. Analyses between 2 groups were performed using the 2-tailed *t* test using GraphPad Prism 8. Data were considered statistically significant at *P <* 0.05. Statistical comparisons between multiple groups were performed by 1-way or 2-way ANOVA, and post hoc analysis was done using Tukey’s multiple-comparisons test, employing GraphPad Prism 8 software. *P <* 0.05 was considered significant.

### Study approval

The Institutional Animal Care and Use Committees of the University of Texas Health and South Texas Veterans Health Care System approved the animal experiments.

## Author contributions

BSK supervised the project in its conception, design, and data interpretation and wrote the manuscript. HJL and MMM conducted experiments, with the aid of SBO, AD, and CSR. LN and TB performed insulin assays. CRK, IR and MT provided genetically modified mice. SEH helped with the generation of KPTIRKO mice. MMM, LN, GGC, DF, MAV, KS, CRK, and RAD participated in data interpretation and writing of the manuscript. All authors provided input and approved the manuscript.

## Supplementary Material

Supplemental data

## Version 1 01/05/2021

In-Press Preview

## Version 2 02/08/2021

Electronic publication
