# Supplementary material for: Proximal tubular epithelial insulin receptor mediates high-fat diet–induced kidney injury
Source: JCI Insight. 2021 Feb 8;6(3):e143619. doi: 10.1172/jci.insight.143619 (PMC7934847; doi:10.1172/jci.insight.143619)
Supplement: Supplemental data [file jciinsight-6-143619-s103.pdf]

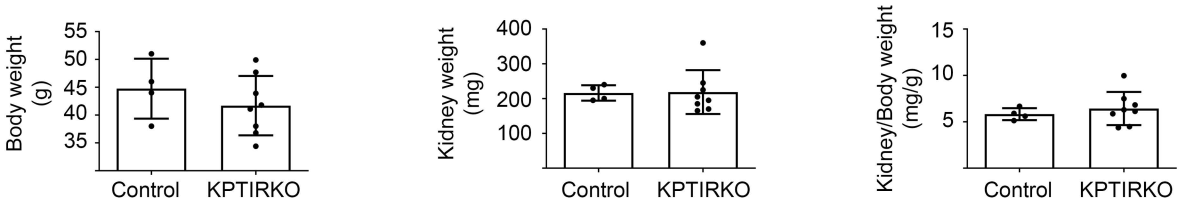

Control (n=4)	KPTIRKO (n=8)
37.25 ±3.5	34.2 ±3.1

Control (n=4)	KPTIRKO (n=8)
216.3 ±22.1	218.8 ±63.1

Control (n=4)	KPTIRKO (n=8)
5.83 ±0.64	6.43 ±1.78

Suppl. Fig. S1. Body weight, kidney weight and kdiney to body weight measurements were made in Control and KPTIRKO mice. There were no differences between the groups in these parameters by t-test. Each dot refers to an individual mouse. Data are presented as mean ± SD.

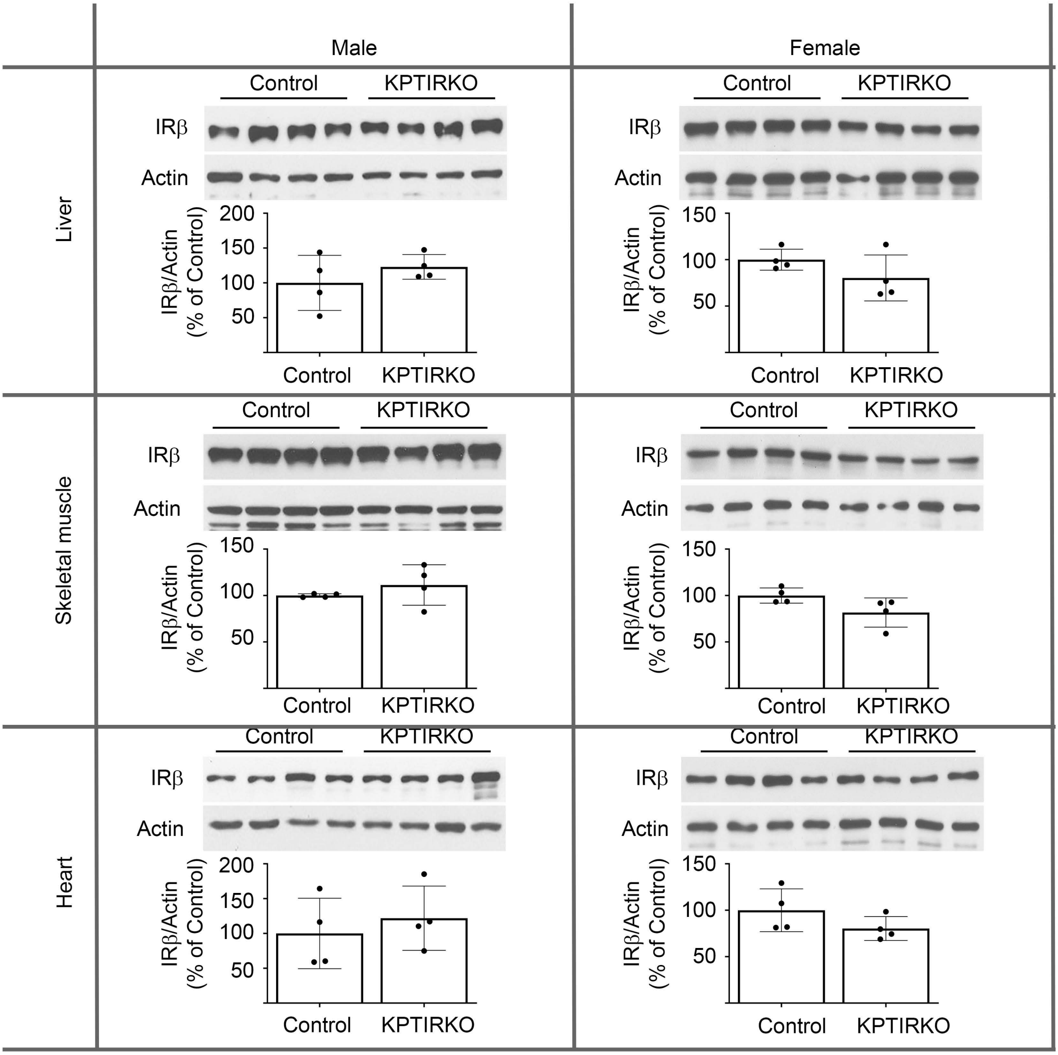

Male
Female
Liver
Skeletal muscle
Heart
Control
KPTIRKO
IRβ
Actin
IRβ/Actin (% of Control)
200
150
100
50

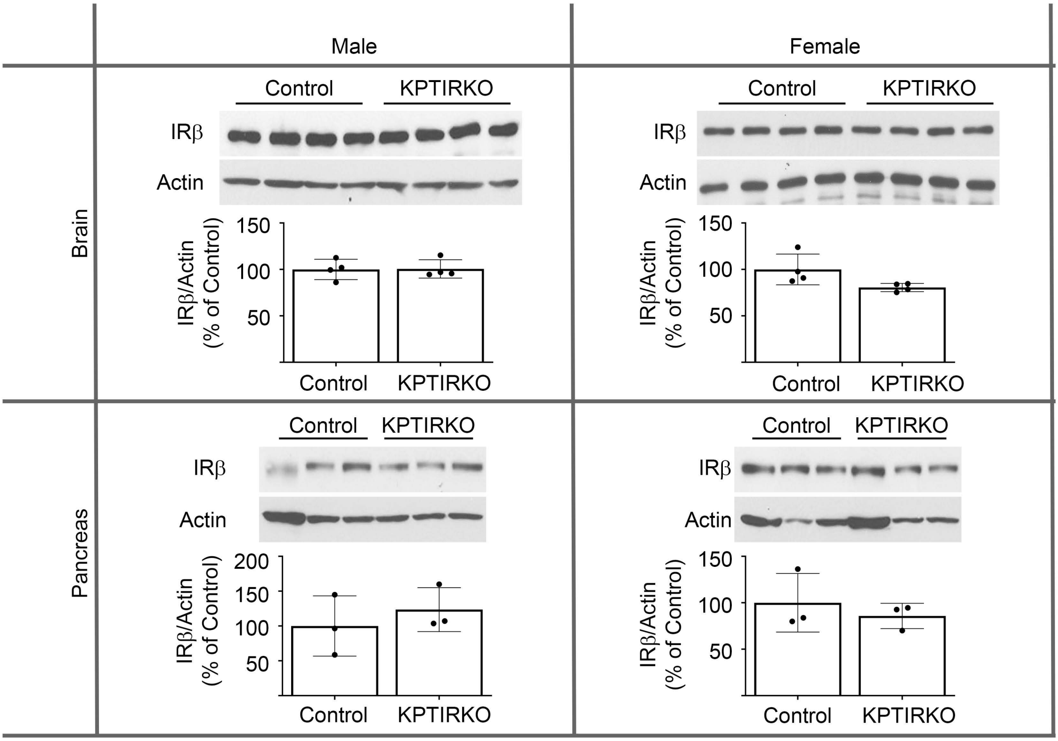

Suppl. Fig. S2. Immunoblotting was performed to assess changes in insulin receptor β (IRβ) expression in different tissues. There were no differences in IRβ expression in the tissues shown between Control and KPTIRKO mice in either male or female mice by t-test. In the graphs, each dot represents one mouse. Data are presented as mean ± SD (n=3-4 mice per group).

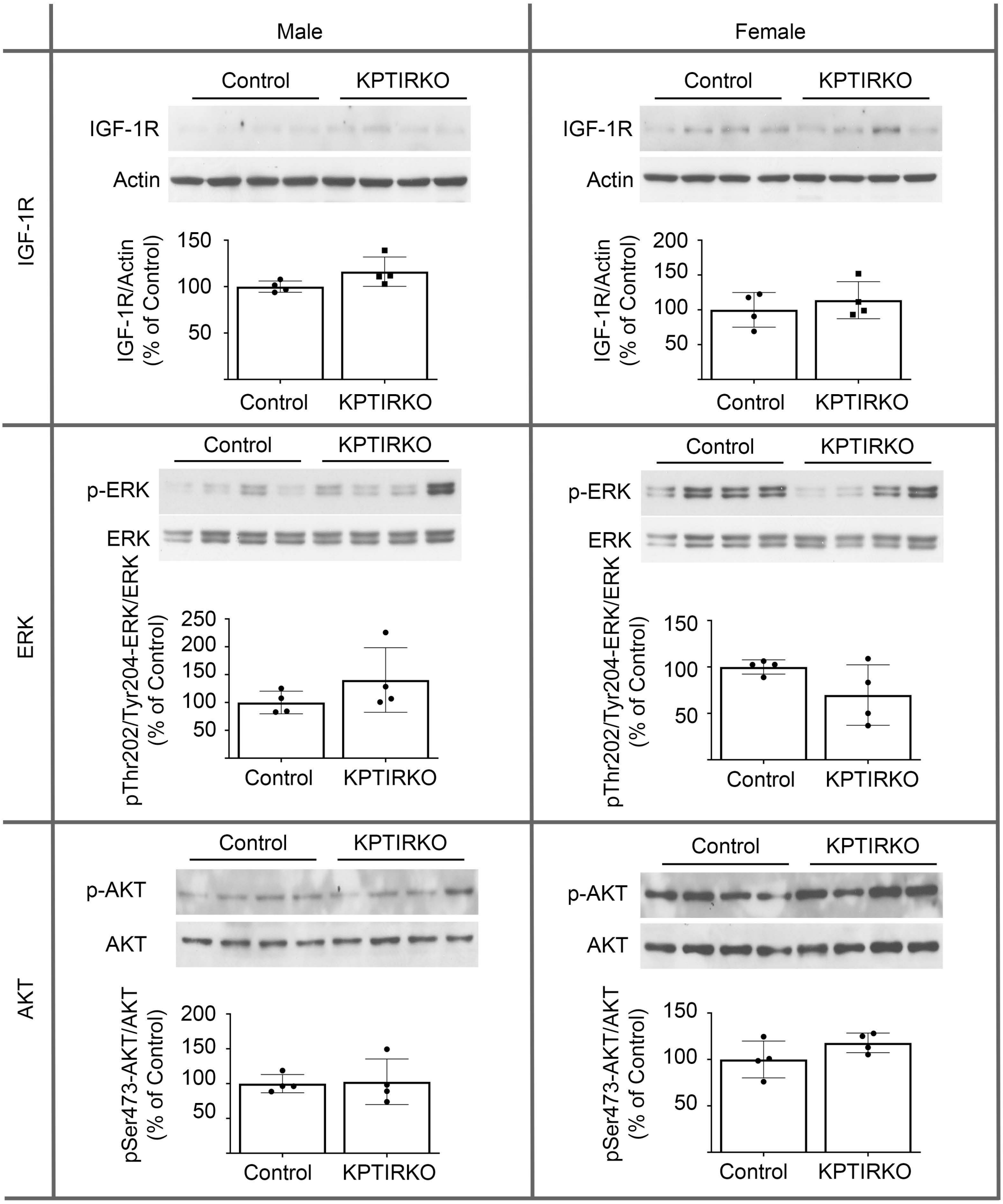

Suppl. Fig. S3. Immunoblotting was performed to assess changes in IGF1 receptor (IGF-1R) expression, phosphorylated Erk, Akt, in renal cortical extracts. There were no differences in the parameters shown between Control and KPTIRKO mice in either male or female mice by t-test. In the graphs, each dot represents one mouse. Data are presented as mean ± SD (n=4 mice per group).

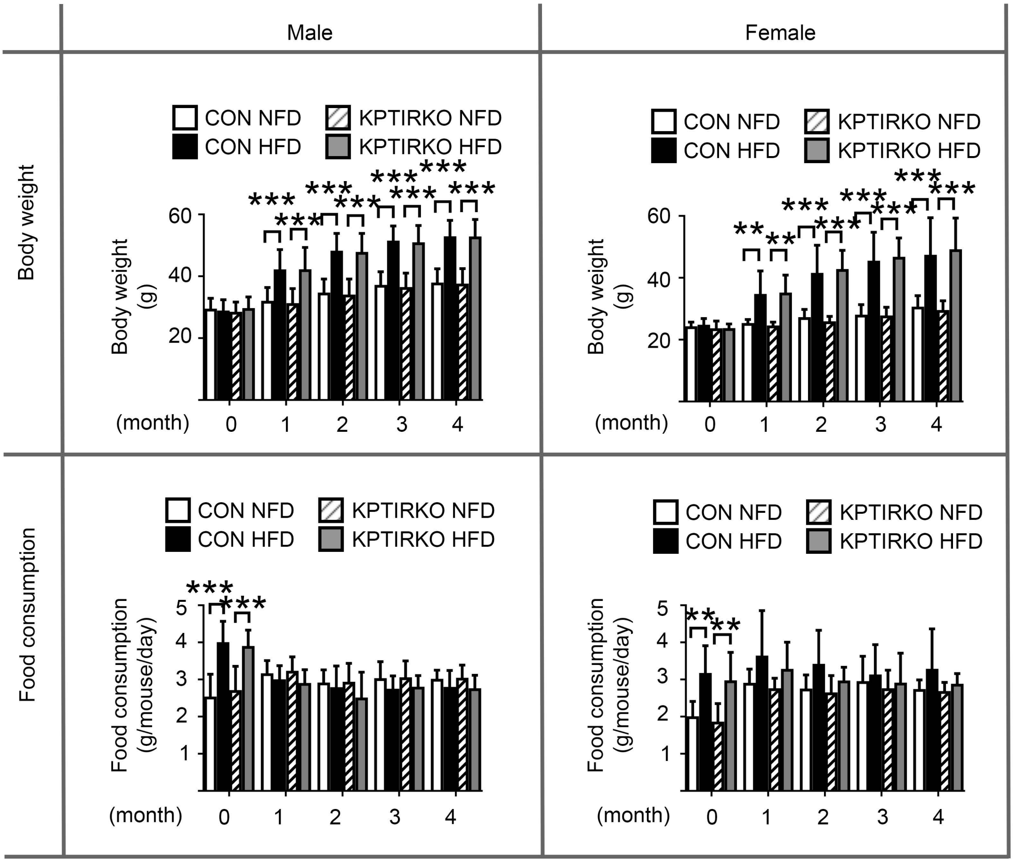

Suppl. Fig. S4. Body weights and food consumption of mice were measured every month. There were no differences between Control and KPTIRKO mice in either male or female mice on NFD or HFD. Data are presented as mean ± SD. $^{**}p<0.01$, $^{***}p<0.001$ by two-way ANOVA (Male, n=14-15 mice per group; Female, n=7-9 mice per group).

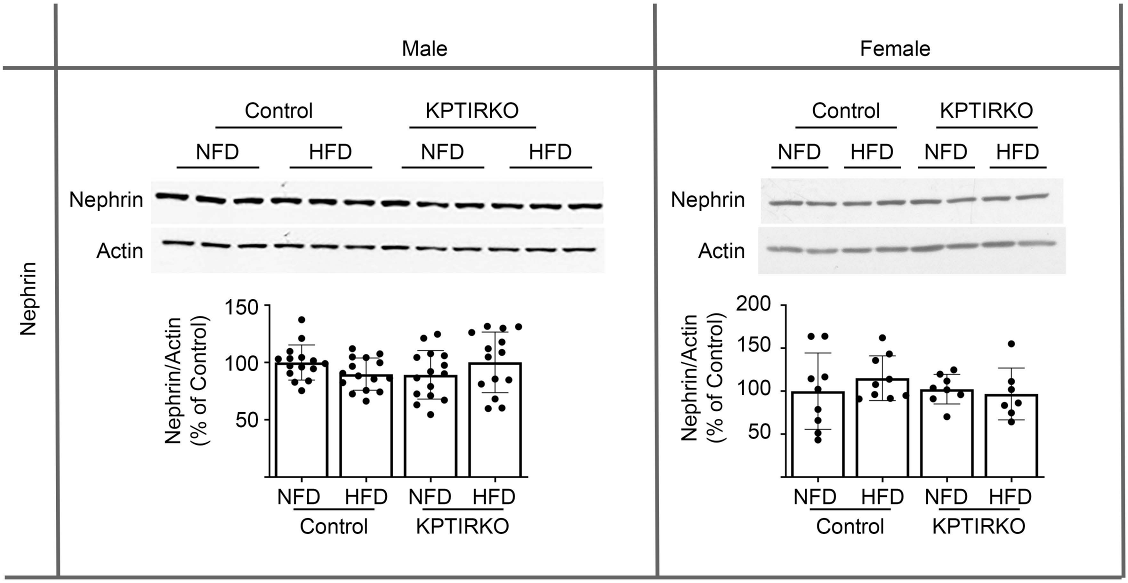

Suppl. Fig. S5. Immunoblotting showed that nephrin expression was unchanged (by one-way ANOVA) following HFD in male and female mice of both genotypes (Male, n=14-15 mice per group: Female, n=7-9 mice per group).

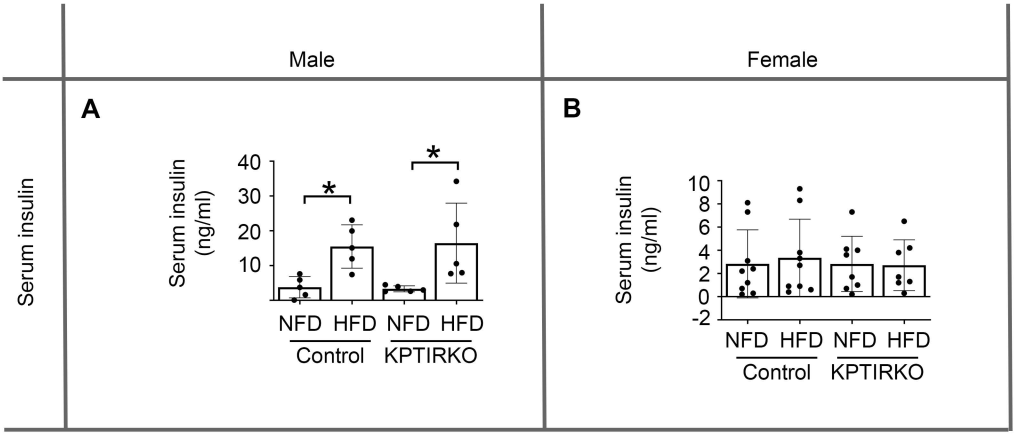

Suppl. Fig. S6. A. On NFD, fasting serum insulin levels were similar in male control and KPTIRKO mice (n=5 mice per group); HFD resulted in hyperinsulinemia in both Control and KPTIRKO mice to the same extent. **B.** There was no difference in random serum insulin levels among the four groups of female mice (n=7-9 mice per group). Data are presented as mean ± SD. *p<0.05 by one-way ANOVA

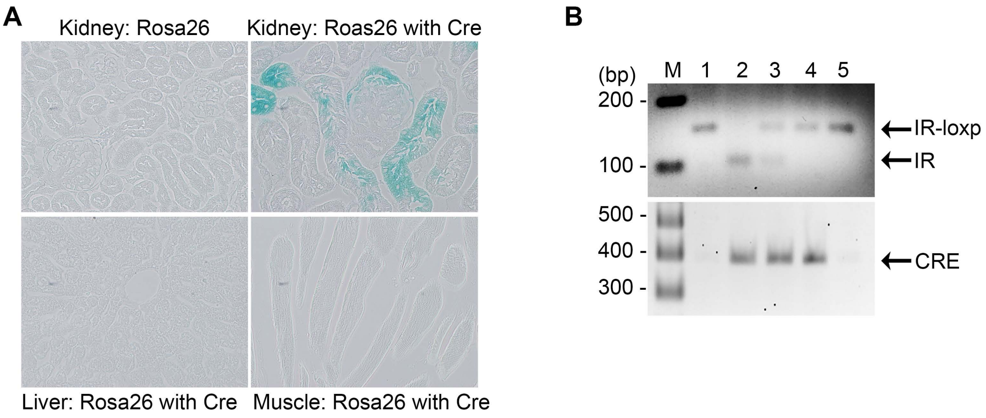

Suppl. Fig S7. Generation of kidney proximal tubule IR knock out mice using the Sglt2 promoter. A. Sglt2 Cre mice were crossed with Rosa26 reporter mice designed to express LacZ; in the hybrid mice β-galactosidase activity detected by X-gal staining was seen only in the proximal tubules of the kidney and not in the other parts of nephron or other organs. **B.** Genotyping of DNA obtained from tails: PCR was performed using primers for IR loxP, IR and Cre. M: Standard markers, lane 1: IR loxp breeder, 2: Sglt2-Cre breeder, 3. Heterozygous KPTIRKO mouse, 4. Homozygous KPTIRKO mouse, 5. Homozygous IR loxp mouse (Control).

Supplementary Table 1. Clinical parameters in Control and KPTIRKO mice

	Control (n=4)	KPTIRKO (n=5-6)
BUN (mg/dL)	20.5 ±10.5	21.3 ±3.7
Serum creatinine (mg/dL)	0.3 ±0	0.3 ±0
